# Supplementary material for: Detection of anti-correlation of hot and cold baryons in galaxy clusters
Source: Nat Commun. 2019 Jul 2;10:2504. doi: 10.1038/s41467-019-10471-y (PMC6606644; doi:10.1038/s41467-019-10471-y)
Supplement: Supplementary file 1 — Supplementary Information [file 41467_2019_10471_MOESM1_ESM.pdf]

SUPPLEMENTARY INFORMATION

DETECTION OF ANTI-CORRELATION OF HOT AND COLD BARYONS IN GALAXY CLUSTERS

ARYA FARAHI[1,2], SARAH L. MULROY[3], AUGUST E. EVRARD[1,4], GRAHAM P. SMITH[3], ALEXIS FINOGUENOV[5,6], HERVÉ BOURDIN[7,8], JOHN E. CARLSTROM[9], CHRIS P. HAINES[10], DANIEL P. MARRONE[11], ROSSELLA MARTINO[8], PASQUALE MAZZOTTA[7,8], CHRISTINE O'DONNELL[11], AND NOBUHIRO OKABE[12,13,14]

[1]Department of Physics, University of Michigan, Ann Arbor, MI 48109, USA

[2]McWilliams Center for Cosmology, Department of Physics, Carnegie Mellon University, Pittsburgh, PA 15213, USA

[3]School of Physics and Astronomy, University of Birmingham, Birmingham, B15 2TT, England

[4]Department of Astronomy, University of Michigan, Ann Arbor, MI 48109, USA

[5]Department of Physics, University of Helsinki, Gustaf Hällströmin katu 2a, 00014 Helsinki, Finland

[6]Max-Planck-Institute for Extraterrestrial Physics, Giessenbachstrasse, 85741 Garching, Germany

[7]Harvard Smithsonian Centre for Astrophysics, 60 Garden Street, Cambridge, MA 02138, USA

[8]Dipartimento di Fisica, Università degli Studi di Roma "Tor Vergata", via della Ricerca Scientifica 1, 00133, Roma, Italy

[9]Kavli Institute for Cosmological Physics, Department of Astronomy and Astrophysics, University of Chicago, IL 60637

[10]INAF - Osservatorio Astronomico di Brera, Via Brera 28, 20122 Milano, Italy

[11]Steward Observatory, University of Arizona, 933 North Cherry Avenue, Tucson, AZ 85721, USA

[12]Department of Physical Science, Hiroshima University, 1-3-1 Kagamiyama, Higashi-Hiroshima, Hiroshima 739-8526, Japan

[13]Hiroshima Astrophysical Science Center, Hiroshima University, 1-3-1 Kagamiyama, Higashi-Hiroshima, Hiroshima 739-8526, Japan

[14]Core Research for Energetic Universe, Hiroshima University, 1-3-1 Kagamiyama, Higashi-Hiroshima, Hiroshima 739-8526, Japan

Systematic Effects. In this section, we study the systematic effects to support the claims made in the main text. We are primarily interested in constraining the correlation coefficient between hot gas mass and stellar mass of the underlaying halos population, which is expected to be anti-correlated. We examine here sources of bias which could modify the estimated correlation. Throughout this section we assume power-law for the mean relation between mass and observables with log-Normal scatter, unless otherwise mentioned.

Effect of biased scaling relation slope. One potential systematic error arises from a potential bias in the posterior mean scaling relation parameters relative to the underlying truth. To illustrate this effect, we generate a synthetic sample of clusters with a known input scaling parameters, then estimate the correlation coefficient imposing a biased set of parameters. To start, we take the LoCuSS weak-lensing masses and assume a set of $M_{\rm gas}$–$M_{\rm WL}$ and $L_{K,\rm tot}$–$M_{\rm WL}$ relations. For each cluster, we draw a random $M_{\rm gas}$ and $L_{K,\rm tot}$ from a multivariate log-normal distribution with a set of input correlation coefficients and 20% intrinsic scatter. Cluster residuals about the mean relation are estimated assuming a set of biased scaling relations consistent with ref.[1]. Finally, the correlation coefficient is estimated with Equation 3 in the Methods section. For each input correlation coefficient, 1,000 realizations of LoCuSS-like cluster sample are generated. Figure 1 illustrates the shift in the estimated correlation coefficient as a function of input correlation coefficient.

While the correlation coefficients based on the gas mass, $M_{\rm gas}$, support negative values for both $L_{K,\rm tot}$ and λ, but the statistical significance is smaller than X-ray luminosity, $L_{X,\rm ce}$, and stellar mass proxies. In the companion paper, we note that the slope of the $M_{\rm gas}$ scaling with halo mass is 0.77 ± 0.1, lower than values above one derived by previous observational studies[2] and from modern hydrodynamic simulations[3], which yield values slightly above unity. The purple line in Figure 1 shows that a bias of 0.3 in $M_{\rm gas}$ slope would produce an underestimate in the anti-correlation magnitude, potentially helping to explain why $L_{X,\rm ce}$ provides more significant evidence for anti-correlation than $M_{\rm gas}$.

Effect of extrinsic or underestimated statistical errors. Our likelihood model assumes that statistical errors of each property are accurate and that the remaining residuals about the mean scaling relation reflect only intrinsic scatter of the underlying halo population that host the clusters in the LoCuSS sample. Properties that are subject to extrinsic contributions not already incorporated into the statistical error budget represent another potential source of systematic error. For example, a recent study shows that the uncertainties quoted for λ by the redMaPPer algorithm underestimate the extrinsic scatter driven by projected galaxies lying outside the host halo[4].

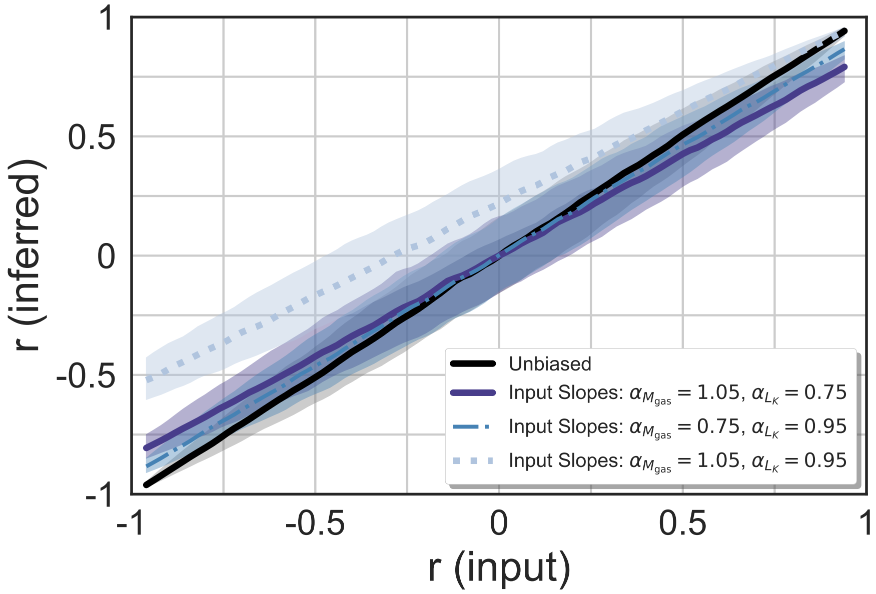

Supplementary Figure 1. **The effect of underestimating the slope.** Sensitivity test of the estimated property correlation for synthetic samples patterned after the LoCuSS cluster sample. A posterior slope of 0.75 for both $M_{\rm gas}$–$M_{\rm WL}$ and $L_{K,\rm tot}$–$M_{\rm WL}$ relations is assumed, while the input true slope takes the values specified in the legend. The shaded areas are 68% confidence intervals derived from 1,000 realizations for each input correlation coefficient.

We test the effect of such systematics by realizing synthetic samples of pairs of properties, $\{M_{\rm star},\, M_{\rm gas}\}$, subject to additional, uncorrelated sources of uncertainty. We assume $M_{\rm star}$–$M_{\rm WL}$ and $M_{\rm gas}$–$M_{\rm WL}$ relations with unit mean slopes and a two-dimensional, log-normal probability distribution with 20% intrinsic scatter in each component and an input correlation coefficient, r. Each halo property is then further perturbed with uncorrelated scatter in each component, of variable magnitude $\sigma_{\rm star}$ and $\sigma_{\rm gas}$, to obtain the observed quantities. For each input correlation, we generate 1,000 realizations of 41-object samples, measuring the property correlation between the observed quantities with our likelihood model. Figure 2 illustrates that such extrinsic scatter leads to inferred correlations that underestimate the magnitude of the underlying population. We note that the difference in posterior correlation coefficient estimates for the $\{M_{\rm gas},\, L_{K,\rm tot}\}$ pair and $\{M_{\rm gas}, \lambda\}$ pair seen in Figure 1 in the Results section could be understood if λ is subject to a larger extrinsic scatter effect than $L_{K,\rm tot}$.

The effects of bias in inferred slope and uncalibrated scatter can explain the systematic shifts in the marginalized posterior distributions seen in Figure 1 in the Results section. With current data, we are not able to assess the contribution of each systematic effect. For example, whether the richness is a noisier measurement of true halo stellar mass with respect to $L_{K,\rm tot}$, or whether the richness measurement uncertainties are underestimated, or both, cannot be addressed with the existing observational data. Detailed simulation and future observational studies are needed to calibrate and understand these effects.

Orientation effects. Massive halos deviate from spherical symmetry, with collisionless components tending toward prolate ellipsoidal shapes with minor to major axis ratio of 0.6 [5]. The collisional nature of the hot gas drives it to a more spherical shape formed by equipotential surfaces. It is reasonable to ask whether correlations between weak-lensing mass and stellar properties driven by orientation might induce an anticorrelation with hot gas properties. To address this question, we apply the log-normal population model of [6] under the assumption of moderate to strong covariance between weak-lensing mass and total K-band light at fixed true mass, and zero intrinsic correlation of either of these properties with hot gas properties. We show here that the correlation of hot gas and K-band luminosity conditioned on weak-lensing mass can be driven to negative values, but only under extreme values of model parameters that are very unlikely.

We consider the likelihood of a vector of properties, $p(\mathbf{s}\,|\,M_{\rm true}, z)$, with elements, $\mathbf{s} = \ln\{M_{\rm WL}, L_k, X\}$ ($X =$ a hot gas property such as $L_X, M_{\rm gas}$, etc.) and let $\mu = \ln M_{\rm true}$. The PDF of true selected mass, $p(\mu\,|\,M_{\rm WL})$, is Gaussian with an assumed width 0.2. Considering the optical properties, L_K or λ, for a sample selected on weak-lensing mass, the correlation between λ (for example) and true halo mass, μ, at fixed weak-lensing mass

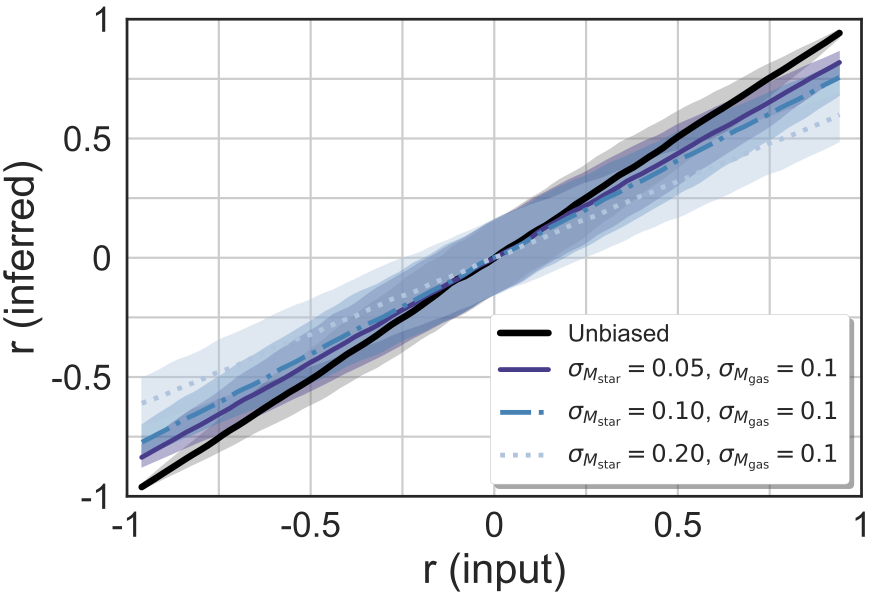

Supplementary Figure 2. **The effect of extrinsic scatter.** Similar to Figure 1, we generate synthetic realizations 41 pairs of intrinsic quantities – $M_{\rm gas}$ and $M_{\rm star}$ – with a known property covariance. We then perturbed intrinsic quantities with extrinsic scatter to obtain measured quantities. The magnitude of extrinsic scatter is specified in the legend. Shaded areas are 68% confidence intervals derived from 1,000 realizations for each input correlation coefficient.

is given by equation (8) of ref.[6],

(1)
$$r_{\ln\lambda,\mu\,|\,M_{\rm WL}} = \frac{\sigma_{\mu\,|\,M_{\rm WL}}/\sigma_{\mu\,|\,\lambda} - r}{\left(1 - r^2 + (\sigma_{\mu\,|\,M_{\rm WL}}/\sigma_{\mu\,|\,\lambda} - r)^2\right)^{1/2}},$$

where $\sigma_{\mu\,|\,M_{\rm WL}} = 0.2$ is the scatter in true mass at fixed weak-lensing mass, $\sigma_{\mu\,|\,\lambda}$ is the similar measure at fixed λ, and r is the correlation coefficient of $\ln M_{\rm WL}$ and $\ln\lambda$ at fixed $M_{\rm true}$. Similar expressions apply for $\lambda \rightarrow L_K$. We assume that the correlation coefficient of weak-lensing-mass and richness is significant, $r0.5$. The above expression indicates that an anti-correlation between true mass and λ or L_K could be induced if the numerator is negative. With the default assumption that a hot gas property, X, is uncorrelated with $M_{\rm WL}$ and λ at fixed true mass, μ, this condition would produce an anti-correlation of λ and X at fixed $M_{\rm WL}$, consistent with our findings.

However, the requirement that Equation 1 be negative implies that the mass scatter at fixed K-band total luminosity must be large in order to suppress the first term in the numerator,

(2)
$$\sigma_{\mu\,|\,\lambda} \geq \sigma_{\mu\,|\,M_{\rm WL}}/r = 0.2/r.$$

A previous work that studied orientation and projection effects of different mass proxies in N-body simulations finds $r = 0.55$ for weak-lensing and red galaxy count[7], meaning $\sigma_{\mu\,|\,\lambda} \simeq 0.4$.

For this effect to be responsible for our findings of a large anti-correlation coefficient between hot and cold gas properties, two factors must conspire: i) $M_{\rm WL}$ and L_K (or λ) at fixed true mass must be very tightly tied, $r \sim 0.9$, and, ii) both optical proxies must have a factor ~ 2 larger scatter in selected true mass compared to $M_{\rm WL}$. Such a strong correlation between weak-lensing mass and optical richness is unlikely, given that random LSS projections across a broad redshift range contribute at least 0.06 to the scatter in $M_{\rm WL}$ [8]. Such a large intrinsic scatter in optical richness for halos above $5 \times 10^{14}\,{\rm M}_\odot$, above which the majority of LoCuSS clusters lie, is not supported by state-of-the-art hydrodynamics simulations[3] and existing observational studies[9].

The following section includes an explicit test that confirms the required conditions.

Line-of-sight projection effect. The cluster properties we use are integrated quantities in the projected space of sky coordinates and photon frequency. Projection of extrinsic material will perturb the intrinsic quantities and, unlike the extrinsic study above, can do so in a correlated manner[7]. Here we model the effect of projection explicitly as a source of extrinsic covariance, $\Sigma_{\rm total} = \Sigma_{\rm halo} + \Sigma_{\rm proj}$. The covariance estimated in this work, Table 2 in the Results section, is the total covariance, and our findings in the main text implicitly assume $\Sigma_{\rm total} \approx \Sigma_{\rm halo}$. Because $\Sigma_{\rm halo}$ and $\Sigma_{\rm proj}$ are degenerate, we cannot disentangle these two components with the observational data in hand.

We perform a set of explicit simulations similar to what we have done above. We generate synthetic realizations of intrinsic quantities of a set of halos with an input property covariance, specified in Table 1 (a), and four models for projection covariance specified in Table 1 (b)-(e). Model (b) is motivated by the fact that the weak-lensing mass and optical observables are expected to be highly correlated [7]. Model (c) is similar to model (b) with larger extrinsic scatter, which illustrates the diluting effect of such a large scatter. It is expected that the optical and weak-lensing observables to be correlated with hot gas observables, but smaller in magnitude [7]. Thus, we consider a third model – model (d) – which includes a small correlation between the X-ray observable and the optical and the mass observables. Finally, model (e) is motivated by our analytical model of the previous section. This improbable model shows that a large extrinsic optical scatter strongly coupled with the mass proxy may indeed induce negative covariance.

Shaded areas are 68% confidence intervals for inferred correlation derived from 1,000 realizations for each input correlation coefficient. The set examines a broad range of scenarios for the projection effect, and our results confirm that negative correlations are diluted. Unless the scatter due to the 2-halo term (projection effect) and correlation between $M_{\rm WL}$ and L_K is very large the projection cannot induce a negative correlation.

As a final test, we revise our inference algorithm to include an explicit projection term with redefining the covariance $\Sigma = \Sigma_{\rm int} + \Sigma_{\rm proj}$. Due to the degeneracy between $\Sigma_{\rm int}$ and $\Sigma_{\rm proj}$, we cannot constrain both quantities simultaneously. We, therefore, employ a fixed $\Sigma_{\rm proj}$, model parameters specified in Table 1 (b), and preform the likelihood to infer $\Sigma_{\rm int}$. We find that $\Sigma_{\rm int}$ posteriors are consistent with our main results. Saying that, it is worth noting that the statistical significance of the anti-correlation between gas mass and K-band luminosity is improved – new p-value = 0.03, and new $r = -0.70^{+0.34}_{-0.20}$.

We do not attempt to correct for this effect as an estimation of such effect is poorly constrained. Estimating the magnitude of this effect requires comprehensive numerical simulations, making synthetic observations, and performing the measurement algorithms which is beyond the scope of this work and is a subject of our future studies. We note that for a larger sample with better statistical power, this effect need to be calibrated and better understood.

Systematics Test Conclusion. The main conclusion drawn from the above tests is that these systematics cannot not induce an anti-correlation signal. Instead, any systematics which is not accounted for or exists in our dataset would reduce the statistical significance of our results and dilute the intrinsic halo anti-correlation signal. Therefore, our results should be interpreted as a conservative lower bound on the magnitude of the anti-correlation between M_{gas} and M_{star} at fixed halo mass. As the quality of data improving and the sample size growing, we need to handle the above systematics better. A future direction should, particularly, address the projection effect for a broad range of observables.

Supplementary Table 1. **The projection models.** The projection covariance models employed in the Supplementary Information to assess the effect of the projections on the inferred anti-correlation signal. σ denotes the scatter parameter and $r_{X,Y}$ denotes the correlation coefficient between X and Y.

Parameter	Input Model	Projection Model 1	Projection Model 2	Projection Model 3	Projection Model 4
σ_M	0	0.10	0.20	0.20	0.20
σ_{L_K}	0.15	0.10	0.20	0.20	0.40
σ_{L_X}	0.15	0.05	0.10	0.10	0.10
r_{M,L_K}	0	0.50	0.50	0.50	0.75
r_{M,L_X}	0	0	0	0.25	0
r_{L_K,L_X}	r (input)	0	0	0.25	0

References

[1] Mulroy, S. L. *et al.* LoCuSS: scaling relations between galaxy cluster mass, gas, and stellar content. *Mon. Not. R. Astron. Soc.* **484**, 60–80 (2019).

[2] Mantz, A. B., Allen, S. W., Morris, R. G. & Schmidt, R. W. Cosmology and astrophysics from relaxed galaxy clusters - III. Thermodynamic profiles and scaling relations. *Mon. Not. R. Astron. Soc.* **456**, 4020–4039 (2016).

[3] Farahi, A., Evrard, A. E., McCarthy, I., Barnes, D. J. & Kay, S. T. Localized massive halo properties in BAHAMAS and MACSIS simulations: scalings, log-normality, and covariance. *Mon. Not. R. Astron. Soc.* (2018).

[4] Costanzi, M. *et al.* Modelling projection effects in optically selected cluster catalogues. *Mon. Not. R. Astron. Soc.* **482**, 490–505 (2019).

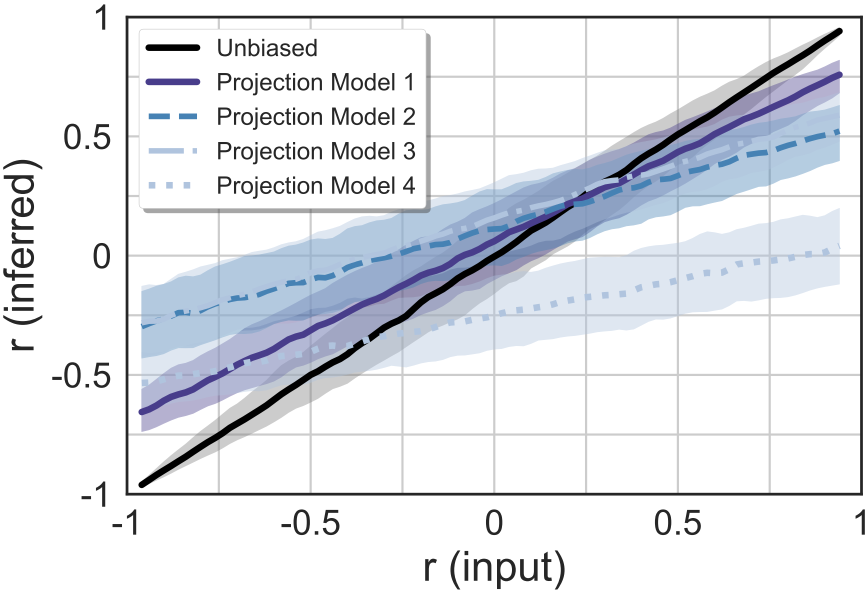

Supplementary Figure 3. **The effect of projection effect.** We generate synthetic realizations of intrinsic quantities of a set of halos with an input property covariance – specified in Table 1 (a) – and a projection covariance – specified in Table 1 (b)-(e) – to obtain measured quantities. The additional random scatter is specified in the legend. Shaded areas are 68% confidence intervals derived from 1,000 realizations for each input correlation coefficient.

[5] Kasun, S. F. & Evrard, A. E. Shapes and Alignments of Galaxy Cluster Halos. *Astrophys. J.* **629**, 781–790 (2005).

[6] Evrard, A. E., Arnault, P., Huterer, D. & Farahi, A. A model for multiproperty galaxy cluster statistics. *Mon. Not. R. Astron. Soc.* **441**, 3562–3569 (2014).

[7] Noh, Y. & Cohn, J. D. Disentangling correlated scatter in cluster mass measurements. *Mon. Not. R. Astron. Soc.* **426**, 1829–1844 (2012).

[8] Hoekstra, H. The effect of distant large scale structure on weak lensing mass estimates. *Astron. Astrophys.* **370**, 743–753 (2001).

[9] Rozo, E. & Rykoff, E. S. redMaPPer II: X-Ray and SZ Performance Benchmarks for the SDSS Catalog. *Astrophys. J.* **783**, 80 (2014).
