# Supplementary material for: Detection of anti-correlation of hot and cold baryons in galaxy clusters
Source: Nat Commun. 2019 Jul 2;10:2504. doi: 10.1038/s41467-019-10471-y (PMC6606644; doi:10.1038/s41467-019-10471-y)
Supplement: Supplementary file 2 — Peer Review File [file 41467_2019_10471_MOESM2_ESM.pdf]

Editorial Note: This manuscript has been previously reviewed at another journal that is not operating a transparent peer review scheme. This document only contains reviewer comments and rebuttal letters for versions considered at Nature Communications.

Reviewer #1 (Remarks to the Author):

The editors of Nature Communications asked me to review the authors' revisions in response to Referee #2's comments. Those revisions are substantive and have changed the tone of the paper, improving it and making it more coherent. Its new title more accurately represents the primary finding. They comment on potential weaknesses of their selection model, and provide support for it in the Supplementary Material. An unexpected (in the context of the authors' hypothesis) anti-correlation between X-ray temperature and stellar mass is still there in the data but should not be a showstopper. I recommend publication.
